# Treatment of perioperative hypertension: Is clevidipine the answer?

**DOI:** 10.4103/1658-354X.57871

**Published:** 2009

**Authors:** Joseph D. Tobias

**Affiliations:** *Departments of Anesthesiology and Pediatrics, University of Missouri, Missouri, Colombia, Missouri E-mail: tobiasj@health.missouri.edu*

Various factors may be responsible for hypertension during the perioperative period, including pain and sympathetic responses during surgical stimulation. Critical times, when stimulation may be maximal and hypertension is present, include laryngoscopy for endotracheal intubation and emergence from anesthesia with the added stimulation from the presence of an endotracheal tube. In addition to blood pressure changes related to perioperative issues, patients with previously unrecognized hypertension or those who are noncompliant with their current medication regimen may present for operative procedures. These issues are further confounded by the current recommendations to hold certain antihypertensive medications (angiotensin converting enzyme inhibitors or angiotensin receptor blockers) for 12-24 hours prior to surgery, given their association with profound and refractory hypotension during anesthetic care.[1,2]

Various complications may be related to perioperative hypertension, including myocardial ischemia, cerebrovascular insults, bleeding, and renal failure. The potential association between perioperative hypertension and an increased risk of complications and mortality was first noted by Smithwick and Thompson in 1953, when they reported that the mortality of hypertensive patients undergoing sympathectomy was six-fold higher than that of normotensive patients undergoing similar operations.[3] This association has subsequently been supported by numerous other studies.[4–8] A cohort of 83,000 patients in the National Veterans Administration Surgical Risk study noted that hypertension was the second most common risk factor for surgical morbidity.[4]

Although elective cases may be delayed until appropriate therapy is instituted and the blood pressure is controlled, the anesthesia provider may be confronted with a hypertensive patient requiring an urgent or emergent surgical procedure. Additionally, overzealous requirements for blood pressure control may result in the unnecessary cancellation or delay of surgical cases. Therefore, rapidly acting and titratable intravenous medications are needed to allow for the perioperative control of blood pressure. Depending on the scenario encountered and the anticipated duration of blood pressure control that is required, anesthesia providers generally use short-acting vasodilators (sodium nitroprusside or nitroglycerin) by continuous infusion or the intermittent administration of longer acting β-adrenergic antagonists, such as, labetolol.

Sodium nitroprusside (SNP) remains one of the most commonly used agents for the acute management of perioperative hypertension. It is a direct-acting, nonselective, peripheral vasodilator that dilates resistance and capacitance vessels to decrease systemic vascular resistance and preload. Its popularity in the perioperative period relates to its rapid onset of action (approximately 30 seconds), rapid peak hypotensive effect (within two minutes), and rapid offset. SNP's physiological effects result from the release of nitric oxide (NO - formerly endothelial-derive relaxant factor), which activates guanylate cyclase, leading to an increase in the intracellular concentration of cyclic guanosine monophosphate (GMP). Cyclic GMP decreases the availability of intracellular calcium by decreasing its release from the sarcoplasmic reticulum into the intracellular space of smooth muscle cells. Despite its efficacy in controlling perioperative blood pressure, SNP's adverse effect profile remains worrisome to many clinicians. Adverse effects may include cerebral vasodilation and an increase in intracranial pressure (ICP) in patients with altered intracranial compliance and light sensitivity, mandating shielding of the bag and infusion tubing from ambient light, increased intrapulmonary shunt due to ablation of hypoxic pulmonary vasoconstriction, evidence of platelet dysfunction from *in vitro* studies, activation of the sympathetic nervous system with reflex tachycardia, rebound hypertension with discontinuation of its administration, tachyphylaxis with prolonged use, cyanide and thiocyanate toxicity, and cardiovascular effects (tachycardia, increased contractility, and decreased diastolic blood pressure), which may shift the myocardial oxygen delivery/demand ratio.[9–11] Furthermore, the potential for excessive hypotension mandates intra-arterial blood pressure monitoring with its use. The other nitrosovasodilator, nitroglycerin (NTG), is primarily used in the perioperative period, to treat coronary ischemia. Its mechanism of action is similar to that of SNP, with the production of nitric oxide. NTG is a direct acting vasodilator that primarily dilates capacitance vessels, reducing venous return with concomitant reductions in stroke volume and cardiac output. It is a short-acting agent with a rapid onset of action (1-2 minutes), a brief duration of action (3-5 minutes) following a bolus dose, and a plasma elimination half-life of 1.5 minutes. As a direct acting vasodilator, NTG shares many of the adverse effects of SNP, including the potential to increase ICP in patients with altered intracranial compliance and inhibition of hypoxic-pulmonary vasoconstriction. Most importantly, given that its primary effect is on the capacitance vessels, NTG has a limited role in the perioperative control of blood pressure.[12,13]

Given the issues with both SNP and NTG, many anesthesia providers choose intermittent dosing of a β-adrenergic antagonist to treat perioperative hypertension. In many cases, as the stimulus causing hypertension is short-lived, intermittent dosing provides an effective alternative for the treatment of alterations in blood pressure. Labetolol is a competitive antagonist at α_1_, β_1_, and β_2_ adrenergic receptors. Blood pressure control results from the blockade of α_1_ and β_1_ adrenergic receptors, with a decrease in both cardiac contractility and heart rate as well as peripheral vasodilatation. This is clinically seen as a decrease in both cardiac output and systemic vascular resistance. Myocardial oxygen consumption decreases as a result of a decrease in afterload, heart rate, and contractility. With intravenous administration, the onset of action is in 5-10 minutes, with the duration of action of 2-4 hours. In addition to its use by intermittent bolus dosing, it may be used in a continuous infusion of 0.5-2 mg/min. Advantages of labetolol include limited effects on intracranial pressure, cerebral blood flow, and cerebral oxygenation, thereby making it a favorite agent in the areas of neuroanesthesia and neurocritical care. Orlowski *et al*. demonstrated a decrease in intracranial pressure after substituting labetolol for SNP.[14] It has limited effects on the intrapulmonary shunt and oxygenation as it does not inhibit hypoxic pulmonary vasoconstriction.

Despite its advantageous properties, there are specific adverse effects and contraindications that may limit the widespread intraoperative use of labetolol. These adverse effects, which are primarily related to its β-adrenergic antagonistic properties, include heart block, heart failure, and bronchospasm. Its potential deleterious effects on airway reactivity may limit its use in patients with asthma or chronic obstructive pulmonary disease. Additionally, unlike many of the other agents that are available for the perioperative control of blood pressure, the half-life of labetolol is significantly longer, thereby occasionally resulting in prolonged hypotension.

The calcium channel antagonists are a structurally diverse group of compounds with a common cellular mechanism of action. Although they have been used most commonly to treat cardiovascular disturbances, such as, angina and essential hypertension, specific agents in this class, such as nicardipine, have also been used via continuous intravenous infusion to control blood pressure in the Operating Room and in the Intensive Care Unit. The calcium channel antagonists interfere with the transmembrane movement of calcium by interacting with either the inner or outer gate of the calcium channel. Although the exact physiological effects may differ from agent to agent, their general physiological actions include vasodilatation and a decrease in the force of cardiac contractility. Verapamil and diltiazem have negative chronotropic and dromotropic effects, while nifedipine and nicardipine predominantly affect the resistance vessels, resulting in vasodilatation.

Nicardipine is a 1, 4 dihydropyridine derivative that vasodilates the systemic, cerebral, and coronary vasculature, with limited effects on myocardial contractility and stroke volume.[15] As nicardipine does have some intrinsic negative chronotropic effects, there is limited rebound tachycardia when compared with SNP and other direct acting vasodilators. Nicardipine has been seen to be an effective agent in various perioperative situations as well as in the control of acute hypertensive crisis in the Intensive Care Unit and the Emergency Room.[16,17] Bernard *et al*. compared the efficacy of SNP and nicardipine in 20 adult patients during isoflurane anesthesia for spinal surgery.[16] An initial dose of nicardipine of 6.2 ± 0.9 mg/hour was required to achieve a mean arterial pressure of 55-60 mmHg, while the infusion requirements varied from 3 to 5 mg/hour. Unlike SNP, no change in arterial oxygenation was seen with nicardipine, suggesting that it may have minimal effects on hypoxic pulmonary vasoconstriction. The efficacy of nicardipine compared favorably with that of SNP. One issue that was noted with nicardipine was a prolonged effect following discontinuation of the infusion. The blood pressure effect persisted for a mean of 43 minutes with a range of 27 to 88 minutes following discontinuation of the infusion.

In this issue of the *Saudi Journal of Anasthesia*, we present an anecdotal experience with the use of clevidipine to control blood pressure during general anesthesia, for an aneurysm coiling in a cohort of three adult patients. We found that clevidipine effectively controlled blood pressure intraoperatively and during emergence from general anesthesia, with only a modest reflex increase in heart rate of 8-10 beats/minute. Clevidipine was recently released by the United States Food and Drug Administration for blood pressure control in adults. It is an ultra-short-acting, calcium channel antagonist of the dihydropyridine group, which provides selective arteriolar vasodilatation.[18,19] Similar to esmolol, it is rapidly metabolized by red blood cells and tissue esterases, resulting in a half-life of 2-3 minutes, which is unaffected by alterations in the renal or hepatic function.[20] Clevidipine reduces blood pressure through a direct and selective effect on arterioles, thereby, reducing the afterload without affecting the cardiac filling pressures and with limited reflex tachycardia. Due to the decrease in systemic vascular resistance, stroke volume and cardiac output usually increase. Despite a decrease in diastolic blood pressure, clevidipine increases the coronary blood flow, indicating that the drug is a direct coronary vasodilator. Clevidipine has been shown to be effective for the treatment of acute hypertensive emergencies as well as preoperative, intraoperative, and postoperative hypertension.[21–23] In these scenarios, its efficacy in controlling blood pressure has been shown to be greater than nitroglycerin and equal to nicardipine and SNP. Data from at least one trial has shown improved survival outcomes with clevidipine when compared with SNP.[22] In addition, when compared with SNP, clevidipine does not decrease the preload and is associated with less tachycardia.[23] Given these hemodynamic effects, its short half-life and easy titratability, and limited adverse event profile, it appears that clevidipine offers another option for the perioperative control of blood pressure.

Clevidipine is supplied in a concentration of 0.5 mg/mL in 50 or 100 mL vials. On account of solubility issues, it is provided in a lipid solution and is contraindicated in patients with allergy to eggs, egg products, soy beans or soy products, as well as, disorders of lipid metabolism. To date, no issues with lipid emulsion have been noted during short-term infusions. Although pricing varies according to region and supplier, the 100 mL vial is approximately $80-100 in the United States. Given its efficacy in the adult cardiac population, future trials in various patient populations, both in the Operating Room and the Intensive Care Unit appear warranted.
